# Animal Heat

**Published:** 1845-05

**Authors:** 


*Animal Heat.*—An extensive series of observations has been made by M. Roger on the temperature, of children in health and various diseases.

In nine examinations of infants from one to twenty minutes after birth, the temperature, (observed in these and in all other cases, in the axilla,) was from 99-95 to 95-45. Immediately after birth the temperature was at the highest; but it quickly fell to near the lowest of those above stated; but, by the next day, it was again completely or nearly what was before. The rapidity of the pulse and of respiration appeared to have no certain relation to the temperature.

In thirty-three infants of from one to seven days old, the most frequent temperature was 98-6; the average was 98-75; the maximum (in one case only;) 102-2; the minimum, (also observed only once 96°-8. All the infants were healthy. The frequency of resperation had no evident or constant relation to the temperature. A few of the infants were of weakly habit; their average was 97-7, the others were strong, and their average temperature was 99°-534. The age of the infant (in this short period,) had no influence on its temperature; neither had its sex, nor its state of sleep or waking, nor the period after sucking.

In twenty-four children, chiefly boys, four months to fourteen years old, the most frequent temperture was above 98°-6; the average was 98°-978; the minimum was 98°-15; the maximum 99°-95. The average temperature of those six years old or under, was 98°-79; of those above six years old, 99°-158. The average number of pulsations in the minute was in those under 6 ears old 102; in those above that age 77; yet the temperature of the latter was higher than that of the former or of younger infants. There was no evident relation between the temperature and the frequency of respiration; nor, in a few examinations, was the temperature affected in a regular way, by active exercise for a short time, or by the stage of digestion.

As already said, in all the examinations from which these results were obtained, the thermometer was held in the axilla; comparative examinations proved that the temperature of the axilla, (though lower than that of internal organs,) was higher than that of any other part of the skin. Of the other parts examined, the warmest was the abdomen, then in succession, the cavity of the mouth, the bend of the arm, the hands, the feet; of which last, the average temperature, in four examinations was only 87°-35. [These results correspond sufficiently with those obtained by Dr. John Davy.]

In diseased states, (to the illustration of which the greater part of the memoir is devoted,) the temperature of the skin in children may descend to 74°-3, and may ascend to 108°-5. Its range of variation is therefore much greater than in adults, in whom M. Andral found it to vary in different diseases not more than from 95° to 107°6.

Dr. John Davy also has contributed some miscellaneous observations on animal heat. They are made with a thermometer placed beneath the tongue. In eight old men and women, all, with one exception, between eighty-seven and ninety-five years of age, the temperature was 95° or 98°-5; therefore not below the average of persons in like circumstances. But two observations showed, that in exposure to external cold, the temperature was more reduced than in young persons, in one case to 95°, in the other to 96°-5. A few observations were also made on persons working in rooms at a temperature of 92°; in one case the temperature under the tongue was 100°, in another 100°-5; and in a third, with an external temperature of 73°, it was 99°. The same slight variations of the temperature of superficial parts in accordance with changes of external temperature were shown by repeated observations on a healthy man in the different seasons at Constantinople. By moderate exercise the temperature was raised, (but not above the general average,) on the surface of the extremities, and was not affected in the internal parts.

Some interesting observations on the effects of hot moist air upon the body were made by M. Constantin James, at the baths or stoves of Nero, called formerly Posidianæ, near Pozzuoli. The physiological part of his account is, that he had first to traverse a passage, leading to the hot springs, about seven feet high, and three wide, in which the temperature in the upper strata of air was 104°F. in the lower 91°-4. After going along this for about fifty yards, the passage narrowing and winding, the temperature in the different strata became 109°-4, and 98°-6, and was very inconvenient; his pulse increased from 70° to 90 times in the minute. After a few instants, as he went on, his pulse became 120°, his temporal arteries beat forcibly, his respiration was short and panting, his body bedewed with sweat; he was obliged to stop every instant, and put his head to the ground, where he could breathe the least heated air. The temperatnre had now become 118°-4 in the upper, and 111°-4 in the lower strata, and the atmosphere was filled with dense vapour. Still descending towards the spring, the atmosphere became still more suffocating; his head felt bursting, he was utterly exhausted, and had nearly lost his consciousness; his pulse could not be counted. The temperature of the spring was 185°, while that of the atmosphere was 122°. Summoning all his strength, the experimenter made his way back through the passage, in which he had been for nearly a quarter of an hour, and of which the whole length was about 120 yards. On coming into the cold air he nearly fainted, and staggered like one drunk, till he was relieved by bleeding at the nose; but through the evening his pulse was 100, he was feverish, had ringings of the ears, and a kind of creeping in the limbs. After a good night's rest he was completely recovered. The water from the spring was clear, and not charged with any gas; neither was any deleterious gas mingled with the heated air.

M. James compares these observations with those made on rabbits and other animals, by M. Magendie. The chief results of these experiments were as follows: two rabits, of the normal temperature of about 102°-2 were placed in two stoves, one at 212°, the other at 140°; the first died sooner than the second, but the temperature of each at the instant of death was the same, namely 111°-2. And the same experiment often repeated, showed that whatever were the ratio at which the heat was applied, the animal died when this increase of nine degrees was attained. In birds whose normal temperature [in some,] is 111°-2, i. e. the same as that at which mammals die, death ensues upon the same increase of nine degrees of temperature; they die when their blood is at 120°-2.

If when an animal is near dying from the effect of heat, an artery be opened, its blood is as black as that of a vein, it does not congulate, and does not become bright on exposure. Exposed to dry heat, the animal loses much weight by evaporation before it dies; exposed to the moist heat it loses no weight, but dies sooner. In the vapour baths of Nero, M. James was almost suffocated in a temperature of 112°, while in those of Testaccio, in which the air is dry, he was but little discomforted by a temperature of 170°; and this has been confirmed on many animals. The quantity of weight lost by evaporation in hot dry air is directly proportionate, not to the temperature, but to the duration of the exposure, and the rate of loss is the same during all the times. After death, the lungs and heart are found in the contrary state to that seen after death from cold; they are empty from blood, and it is collected and extravasated at the surface of the body.

